# Intraoperative carcinoid syndrome during small-bowel neuroendocrine tumour surgery

**DOI:** 10.1530/EC-18-0324

**Published:** 2018-10-04

**Authors:** Myrtille Fouché, Yves Bouffard, Mary-Charlotte Le Goff, Johanne Prothet, François Malavieille, Pierre Sagnard, Françoise Christin, Davy Hayi-Slayman, Arnaud Pasquer, Gilles Poncet, Thomas Walter, Thomas Rimmelé

**Affiliations:** 1 Department of Anaesthesiology and Critical Care Medicine, Edouard Herriot Hospital, Hospices Civils de Lyon, Lyon, France; 2 Department of Visceral Surgery, Edouard Herriot Hospital, Hospices Civils de Lyon, Lyon, France; 3 Department of Hepatogastroenterology and Oncology, Edouard Herriot Hospital, Hospices Civils de Lyon, Lyon, France; 4 EA 7426 Hospices Civils de Lyon-University Claude Bernard Lyon 1-Biomérieux ‘Pathophysiology of Injury-Induced Immunosuppression’ Pi3, Lyon, France

**Keywords:** carcinoids, neuroendocrine tumour, somatostatin

## Abstract

Only few descriptions of intraoperative carcinoid syndrome (ioCS) have been reported. The primary objective of this study was to describe ioCS. A second aim was to identify risk factors of ioCS. We retrospectively analysed patients operated for small-bowel neuroendocrine tumour in our institution between 2007 and 2015, and receiving our preventive local regimen of octreotide continuous administration. ioCS was defined as highly probable in case of rapid (<5 min) arterial blood pressure changes ≥40%, not explained by surgical/anaesthetic management and regressive ≥20% after octreotide bolus injection. Probable cases were ioCS which did not meet all criteria of highly-probable ioCS. Suspected ioCS were detected on the anaesthesia record by an injection of octreotide due to a manifestation which did not meet the criteria for highly-probable or probable ioCS. A total of 81 patients (liver metastases: 59, prior carcinoid syndrome: 49, carcinoid heart disease: 7) were included; 139 ioCS occurred in 45 patients: 45 highly probable, 67 probable and 27 suspected. ioCs was hypertensive (91%) and/or hypotensive (29%). There was no factor, including the use of vasopressors, significantly associated with the occurrence of an ioCS. All surgeries were completed and one patient died from cardiac failure 4 days after surgery. After preoperative octreotide continuous infusion, ioCS were mainly hypertensive. No ioCS risk factors, including vasopressor use, were identified. No intraoperative carcinoid crisis occurred, suggesting the clinical relevance of a standardized octreotide prophylaxis protocol.

## Introduction

Small-bowel neuroendocrine tumours (SB-NETs) are rare secreting neoplasms. Hormones (serotonin, histamine, bradykinin, prostaglandins and chromogranin-A) released into the systemic circulation can lead to a carcinoid syndrome (CS), which occurs in 10–20% of SB-NET patients. CS is characterized by isolated or associated symptoms such as cutaneous flushing (90%), diarrhoea (80%), abdominal pain (35%), bronchospasm (15%) and/or cardiovascular changes (hypo or hypertension, tachycardia or cardiac insufficiency (30%)) (1, 2, 3). This syndrome can progress to a carcinoid crisis, a life-threatening complication combining severe hemodynamic instability, cardiac arrhythmias and failure and refractory bronchoconstriction (4). Up to 40% of patients develop a carcinoid heart disease, a major cause of morbidity and mortality (5, 6, 7, 8).

Expert groups recommend primary SB-NETs open surgical removal with partial bowel resection and lymphadenectomy even in the metastatic stages (>50% of cases at diagnosis) to prevent local complications and decrease tumour mass (9, 10). They also recommend the use of somatostatin analogues due to their anti-proliferative effect on SB-NETs and their antisecretory effect against CS (11, 12, 13, 14, 15).

The perioperative period is at high risk of intraoperative carcinoid syndrome (ioCS) as many triggering factors are present (4). Hormone release can be caused by stress, hypoxemia, hypothermia, hypo or hypertension, pain, induction of anaesthesia, tumour manipulation and pharmacological agents, including anaesthetic drugs. Furthermore, vasopressor use has been suspected to trigger ioCS by promoting SB-NET hormone release and to result in paradoxical effects (4, 8, 13, 16, 17).

However, only few studies reporting on the clinical presentation of ioCS have been published (18, 19, 20, 21). Thus, the primary endpoint of this study was to describe ioCS. The second aim was to identify risk factors of ioCS in patients operated of SB-NETs, receiving a continuous infusion of intravenous somatostatin analogue started from the preoperative period.

## Materials and methods

After approval by the ethics committee of our institution (Comité de protection des Personnes SUD-EST IV, Lyon), we retrospectively analysed a series of consecutive patients who underwent resection of SB-NET in our institution between January 2007 and December 2015. This study was approved by the national data protection commission (Commission Nationale de l’Informatique et des Libertés, CNIL) on 6 November 2015 (n°15-111). Consents have been obtained from patients after full explanation of the purpose and nature of data used. All patients receiving our local regimen of preoperative continuous octreotide were included. Patients operated for other neuroendocrine tumour location, hepatic metastases only or not receiving our octreotide administration regimen were not included.

### Perioperative octreotide administration

Octreotide was administered 12–48 h prior to surgery by intravenous continuous infusion at a dose of 40 µg/h or 80 µg/h if patient had prior CS, hepatic metastases or carcinoid heart disease. Octreotide was continued during the intraoperative and postoperative periods at the same doses. IoCS were treated by additional intraoperative octreotide boluses (0.5–2 µg/kg) and surgical break.

### ioCS definition

Highly probable ioCS was defined as rapid (onset period ≤5 min) hemodynamic changes (heart rate (HR) or blood pressure (BP)) ≥40%, not explained by surgical or anaesthetic management and regressive ≥20% within 5 min after the octreotide bolus injection; probable ioCS was defined as cases for which manifestations did not meet all criteria of highly probable ioCS (onset period of 5–10 min and/or hemodynamic changes of 20–40% and/or no octreotide bolus and/or no reversibility of ≥20% after octreotide injection and/or confounding factors related to anaesthetic management). Suspected ioCS was detected on the anaesthesia record by an injection of octreotide for a manifestation which did not meet the criteria of highly-probable or probable ioCS.

Median variations of BP and HR from basal state to maximum and then during regression for each type of ioCS were collected. Carcinoid crisis was defined as a life-threatening ioCS refractory to octreotide boluses. Carcinoid crisis includes cardiogenic shock, severe cardiac dysrhythmias, cardiac arrest or bronchospasm refractory to bronchodilators and compromising mechanical ventilation.

### Anaesthetic management

All patients had preoperative assessment, including a transthoracic echocardiography at least 48 h prior to surgery. The day before surgery, premedication with antihistamines or gabapentin was prescribed if anxiety was 6 or more on a 10-point verbal numeric scale. After standard monitoring installation, anaesthesia was induced intravenously with propofol or etomidate, remifentanil and cisatracrium. Anaesthesia was maintained with desflurane delivered by control mode mechanical ventilation (Primus, Drägerwerk AG & Co. KGaA, Lübeck, Germany) and with a continuous infusion of remifentanil. Adequate muscle relaxation was obtained by intravenous cisatracrium boluses according to train-of-four monitoring. Two 16 or 18-G peripheral venous catheters and a triple lumen central venous line were inserted. An arterial catheter was inserted at the discretion of the attending anaesthetist as were transoesophageal Doppler (WAKI^e^ TO, Atys medical, Soucieu-en-Jarrest, France) and Bispectral index (BIS) (BIS, Covidien, Ilc, Mansfield, USA). Warming blankets were always used (3M Bair Hugger therapy, 3M Health Care, St. Paul, USA). Hypotensive episodes were treated by octreotide boluses and, if necessary, by ephedrine (0.10–0.15 mg/kg) or phenylephrine (1–2 µg/kg) boluses and then by norepinephrine intravenous continuous infusion starting at dose at 0.05 µg/kg/h.

### Data collection

The following clinical parameters were recorded: age, gender, physical status ASA score, cardiovascular history (hypertension, cardiac failure), prior CS (flushing, diarrhoea, bronchospasm), prior carcinoid heart disease, hepatic and extra-abdominal metastases, elevated urinary 5-hydroxyindoleacetic acid (5-HIAA), and the prior use of long-acting somatostatin analogues. Anaesthesia management with use of premedication, anaesthetic agents, and vasoactive drugs, as well as the timing of occurrence of ioCS during the surgical procedure, hemodynamic changes during ioCS, the type of surgical procedure and number of SB-NETs on histologic examination were recorded, as was death following carcinoid crisis.

### Statistical analysis

Continuous variables with a Gaussian distribution were reported as mean with standard deviation (s.d.) and compared with the Student *t*-test, whereas continuous variables with a non-Gaussian distribution were reported as median and interquartile range (IQR) and compared using the Mann–Whitney test. Univariate analyses of potential ioCS risk factors were performed. Categorical variables were compared using a *χ* ^2^ or Fisher exact test. A *P* value ≤0.05 was considered as statistically significant.

## Results

A total of 81 patients (42 males) underwent SB-NET surgery during the study period and were included. The mean (s.d.) age was 59 (12) years. The ASA score was 1–2 for 65 patients, and 35 had a previous cardiovascular history. Among those, 49 patients had prior CS (diarrhoea 39, flushing 42, bronchospasm 1); seven patients had carcinoid heart disease and two patients a tricuspid valve replacement one year before abdominal surgery. Fifty-nine patients had hepatic metastases and seven an extra-abdominal location of NET. Urinary 5-HIAA levels were elevated for 40 patients. Octreotide protocol was started 21 (8) hours before surgery and was respected for 64 patients (11 patients had a lower dose).

All patients underwent bowel resection, 79 with ileocolic anastomosis and 78 with lymphadenectomy. The median (IQR) number of tumours removed was 1 (1, 2, 3, 4). Hepatic resection was performed in 12 patients. The mean (s.d.) operative time was 300 (92) min. All tumours were confirmed to be SB-NET. Characteristics of intraoperative anaesthetic management are shown in Table 1. Sixty-five patients had premedication, with antihistamines in most of cases.

**Table 1:** Intraoperative anaesthetic management.

	*n*
Hypnotics	
Etomidate	1
Propofol	80
Opioids	
Remifentanil	81
Ketamine (antihyperalgesic dose)	80
Neuromuscular blocking agent	
Cisatracurium	81
Succinylcholine	2
Postoperative analgesia	
Morphine	75
Lidocaine IVSE	7
Haemodynamic monitoring	
Arterial catheter	52
Transoesophageal Doppler	16
Bispectral index	35
Vasopressor use	
Ephedrine	71
Phenylephrine	28
Noradrenaline	11
Volume expansion (mean ± s.d., mL/kg/h)	
Crystalloid	6.6 ± 2.2
Colloid	0.7 ± 0.9
Albumin	0
Blood transfusion (mean ± s.d.)	2 (2)
Number of packed red blood cells	2.5 ± 0.7
Urinary output (mean ± s.d., mL/kg/h)	1.5 ± 1.1

**Figure 1:**
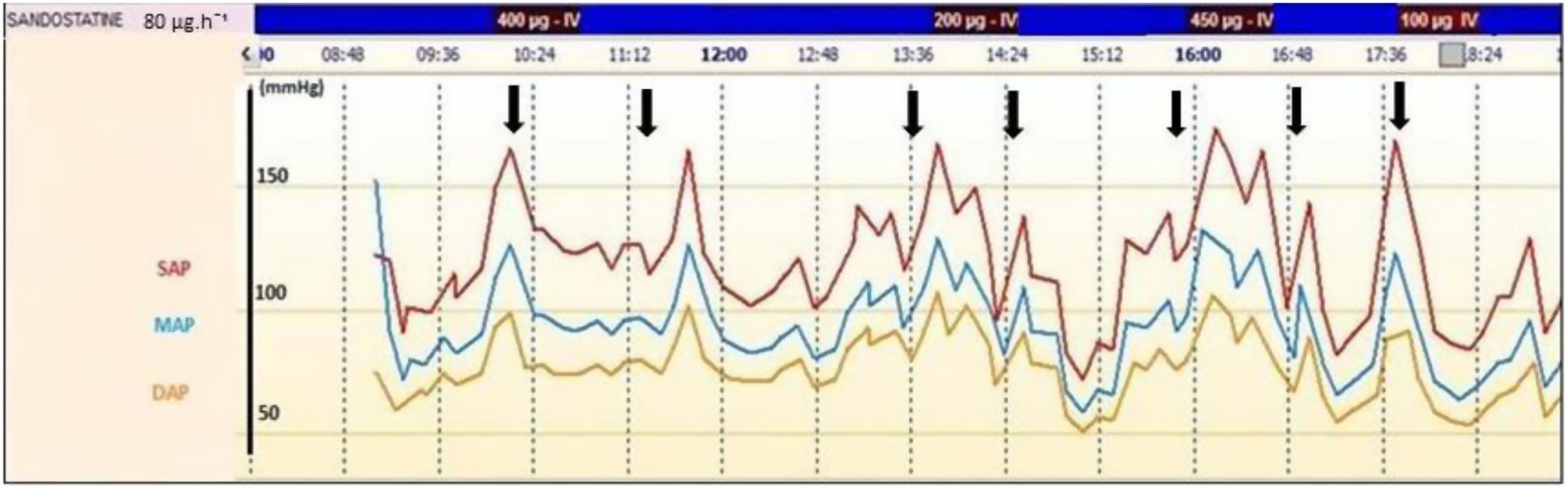
Example of individual recording of multiple intraoperative carcinoid syndromes during small-bowel neuroendocrine tumour surgery.

A total of 139 episodes of ioCS (highly probable, probable and suspected) occurred in 45 patients. The mean (s.d.) number of ioCS episodes per patient was 3 (2) (Fig. 1). Up to 11 ioCS occurred for two patients during surgery. IoCS was a hypertensive (91%, 41 patients, 107 episodes) and/or a hypotensive episode (29%, 13 patients, 32 episodes). Cutaneous flushing occurred in 3 patients. No episode of dysrhythmia or bronchospasm occurred. Time of occurrence of ioCS was tumour manipulation (*n* = 37 patients, 82%), incision (*n* = 8 patients, 18%) and anaesthetic induction (*n* = 2 patients, 4%). No triggering factor of ioCS was identified for 11 (1/4) patients. Patients received a mean (s.d.) of 3 (3) octreotide boluses during surgery. No side effects or cardiac conduction abnormality induced by octreotide injection occurred. BP and HR variation during ioCS were recorded (Table 2). BP changes during hypotensive episodes of ioCS were more pronounced on systolic arterial pressure than on mean and diastolic arterial pressure. There was no significant HR variation during ioCS.

**Table 2:** Hemodynamic changes during hypertensive and hypotensive episodes of intraoperative carcinoid syndrome.

Variations median % (IQR)	Hypertensive episode			Hypotensive episode		
	Highly probable (*N* = 36)	Probable (*N* = 56)	Suspected (*N* = 15)	Highly probable (*N* = 9)	Probable (*N* = 11)	Suspected (*N* = 12)
SAP before ≥ during	53 (44–64)	35 (27–48)	18 (5–33)	49 (46–55)	28 (24–49)	10 (6–15)
SAP during ≥ after	24 (21–30)	22 (14–30)	8 (2–19)	63 (47–81)	28 (21–33)	7 (2–15)
MAP before ≥ during	51 (45–63)	35 (24–51)	14 (4–3)	24 (23–30)	15 (9–19)	3 (2–6)
MAP during ≥ after	31 (22–40)	23 (15–33)	3 (−1 to 12)	49 (34–88)	24 (14–33)	5 (1–15)
DAP before ≥ during	49 (39–59)	35 (22–50)	9 (−1 to 31)	16 (13–18)	9 (6–13)	2 (1–4)
DAP during ≥ after	24 (13–34)	18 (8–24)	1 (−2 to 8)	48 (37–71)	25 (10–34)	5 (−1 to 18)
HR before ≥ during	11 (3–20)	10 (2–16)	1 (−3 to 8)	3 (0–10)	0 (−3 to 1)	−1 (−2 to 0)
HR during ≥ after	3 (0–12)	2 (−1 to 6)	0 (−2 to 7)	−2 (−14 to 1)	2 (−3 to 9)	0 (1–4)

Among probable ioCS, missing criteria to classify them as highly probable ioCS were, for hypertensive and hypotensive episodes respectively, hemodynamic changes ≤40% (57% and 73%), onset period between 5 and 10 min (14 and 9%), no octreotide injection (48 and 0%), reversibility ≤20% after octreotide injection (4 and 0%) and confounding factors with anaesthetic management (16 and 18%).

There was no factor, including the use of vasopressors, significantly associated with the occurrence of ioCS (Table 3). The analysis was repeated with patients who experienced only highly probable ioCS with the same result.

**Table 3:** Characteristics of patients according to occurrence of intraoperative carcinoid syndrome (ioCS).

	No ioCS, *n*	At least one ioCS, *n*	*P*
Number	36	45	
Preoperative carcinoid syndrome	21	28	0.90
Diarrhoea	17	22	0.94
Cutaneous flush	17	25	0.60
Carcinoid heart disease	1	6	0.12
Hepatic metastases	23	36	0.17
Elevated preoperative output of 5-hydroxylindoleacetic acid (missing data = 17)	16	24	0.14
Premedication with antihistamines	28	29	0.29
Intraoperative vasopressors use			
Ephedrine	32	39	1.00
Phenylephrine	12	16	0.98
Noradrenaline	4	7	0.75
Hepatic resection	6	6	0.92

No intraoperative carcinoid crisis occurred during the study period. All surgeries were completed. One patient died postoperatively, 4 days after surgery, developing a carcinoid crisis with a refractory cardiac failure and a cardiac arrest. He was a 74-year-old man who had a highly secreting tumour with a severe clinical presentation, an asymptomatic carcinoid heart disease, and hepatic metastases.

## Discussion

After preoperative continuous octreotide infusion, ioCS were mostly hypertensive and no ioCS risk factor was identified. To our knowledge, this is the first study that pre-defined ioCS in order to describe the occurrence and the risk factors of this complication.

There is no consensus for ioCS definition. Seymour *et al*. underlined the heterogeneity in the definition of ioCS and carcinoid crisis (20). Moreover, clinical manifestations of ioCS under general anaesthesia can be modified and identifying this complication can be difficult. In addition, Bijker *et al.* underlined the difficulty to define intraoperative hypotension regarding the variability of definitions through studies depending on the threshold values, BP type (systolic vs mean BP), measurement interval and method (noninvasive vs invasive) and minimal episode duration (22). It results in wide incidence of intraoperative hypotension and different associations between intraoperative hypotension and adverse outcomes. This suggests that a dynamic approach of intraoperative BP variations could be of clinical relevance, more than dichotomic thresholds. To our knowledge, the present study is the first to analyse ioCS as a dynamic phenomenon using a definition taking into account onset time, variation of BP or HR, reversibility after octreotide injection and absence of confounding factors with surgical or anaesthetic management.

The overall incidence of ioCS was higher than the previously published series (24–38%) (18, 19, 21). However, the analysis of each episode reveals that incidence of ioCS could be lower. Indeed, some suspected ioCS may have incorrectly been recorded as ioCS. Clinical manifestations of ioCS contrast with other studies which report hypotensive episodes to be much more frequent. Indeed, Massimino *et al.* reported a hypotensive episode for 19% of patients, defined as PAS <80 mmHg during ten minutes (19). Condron *et al.* published a series of 127 patients with 30% of ioCS (76% of hypotensive and 7% of hypertensive episodes) (21). One explanation could be the regimen of octreotide administration. There remains variability in practices regarding the timing and dosage of preoperative octreotide. Recommendations regarding octreotide infusion vary according to administration regimen, preoperative dose and infusion rate during surgery (12, 13, 21). Different affinity with somatostatin receptors and a dose-dependent effect on hormone release could explain the different types of clinical manifestations.

Few patients needed several injections (up to three boluses) to control ioCS suggesting the efficiency of low dose of octreotide. No intraoperative carcinoid crisis occurred and all surgeries were completed. Although continuous octreotide infusion is insufficient to prevent all ioCS, our local protocol seems to be effective to prevent carcinoid crisis, the most feared complication.

No factor was statistically associated with occurrence of ioCS. In 1/4 of cases, no triggering factor was found suggesting that ioCS can occur at any time and that anaesthetists should be constantly ready to treat hemodynamic instability. The small sample size is likely to be a major limitation for the identification of predictive factors. However, another explanation could be related to the protocol used that includes a double dose of octreotide in case of preoperative CS, hepatic metastases, or carcinoid heart disease, which may have reduced the risk of ioCS in such patients to that of other patients. Some reports suggest that vasopressor use can trigger ioCS and recommend to avoid this medication (4, 8, 13, 17). This is controversial and many studies have reported safety with vasopressor use (12, 18, 19, 21). The results herein suggest that intraoperative use of vasopressor in CS patients is safe to treat hypotension without paradoxical effects. The results also suggest that systematic premedication with antihistamines to decrease anxiety and stress in order to avoid hormone release is useless.

The study has some limitations. First, it was a single-centre study, but this design allowed for homogeneous surgical procedure and anaesthetic management. These results are related to our local protocol of octreotide and they could be different with other octreotide administration protocols. Second, our definition of ioCS was developed by an experienced anaesthetist team, but it can be discussed. We did not include duration of each ioCS. The analysis of hypertensive episodes reveals an increase of BP ≥50% during highly probable ioCS. This threshold for diagnosis of ioCS could be decreased considering ioCS classified as probable because of a variation of BP lower than 40%. Thus, a better definition of ioCS is needed but requires validation in a prospective multicentric study. Third, the retrospective nature of this study did not allow for a deep assessment of postoperative complications. Thus, the impact of ioCS on outcomes, as Condron and Massimino *et al.* reported was not feasible (19, 21). In addition, a lack of power due to the relatively small sample size did not facilitate the identification of ioCS risk factors.

In conclusion, when a preoperative continuous octreotide infusion is administered to patients, ioCS are mainly hypertensive. No ioCS risk factors, including vasopressor use, were identified. A standardized octreotide prophylaxis protocol seems to be clinically relevant to manage ioCS and to prevent occurrence of carcinoid crisis.

## Declaration of interest

The authors declare that there is no conflict of interest that could be perceived as prejudicing the impartiality of the research reported.

## Funding

This research did not receive any specific grant from any funding agency in the public, commercial or not-for-profit sector.

## Author contribution statement

Contributed to the design of the work, acquisition of data: M F, Y B, M-C L, J P, F M, P S, F C, D H S, A P, G P , T W, T R. Conducted the analysis and interpretation of data: M F, Y B, T W. Drafted the manuscript: M F, Y B. Revised the work for important intellectual content: M-C L, F M, D H S, F C, J P, P S, T W, A P, G P, T R. All authors approved the submitted version of the manuscript.
